# Patients’ Perceived Level of Clinician Knowledge of Transgender Health Care, Self-rated Health, and Psychological Distress Among Transgender Adults

**DOI:** 10.1001/jamanetworkopen.2023.15083

**Published:** 2023-05-25

**Authors:** Gabe H. Miller, Guadalupe Marquez-Velarde, Alex R. Mills, Stephanie M. Hernandez, Lauren E. Brown, Mudasir Mustafa, Jesse E. Shircliff

**Affiliations:** 1 Department of Sociology, University of Alabama at Birmingham; 2 Department of Sociology and Anthropology, Utah State University, Logan; 3 Department of Pharmacy Practice, University of Mississippi School of Pharmacy, Jackson; 4 Center for Gender and Sexual Minority Health, University of Mississippi Medical Center, Jackson; 5 Department of Epidemiology and Biostatistics, Drexel University, Philadelphia, Pennsylvania; 6 Department of Sociology, Mississippi State University

## Abstract

**Importance:**

Transgender, gender nonbinary, and genderqueer people are at increased risk for negative health outcomes, and medical school education is currently lacking on inclusion of these topics. However, there is little evidence of an association of clinician knowledge with the health of transgender people.

**Objective:**

To evaluate the associations of patients’ perceptions of clinician knowledge with self-rated health and severe psychological distress among transgender people.

**Design, Setting, and Participants:**

In this cross-sectional study, a secondary data analysis of the 2015 US Transgender Survey (a survey of transgender, gender nonbinary, and genderqueer adults conducted across 50 states) Washington, DC, US territories, and US military bases in 2015 was performed. Data were analyzed from February to November 2022.

**Exposures:**

Patients’ perception of their clinician’s knowledge about transgender health care.

**Main Outcomes and Measures:**

Self-rated health, dichotomized as poor or fair vs excellent, very good, or good, and severe psychological distress (scoring a validated threshold of ≥13 on the Kessler Psychological Distress Scale).

**Results:**

The sample included a total of 27 715 respondents (9238 transgender women [33.3%; 55.1% weighted; 95% CI, 53.4%-56.7%], 22 658 non-Hispanic White individuals [81.8%; 65.6% weighted; 95% CI, 63.7%-67.5%], and 4085 individuals aged 45-64 years [14.7%; 33.8% weighted; 95% CI, 32.0%-35.5%]). Of 23 318 individuals who answered questions regarding their perceptions of their clinicians’ level of knowledge, 5732 (24.6%) reported their clinician knows almost everything about transgender care, 4083 (17.5%) reported their clinician knows most things, 3446 (14.8%) reported their clinician knows some things, 2680 (11.5%) reported their clinician knows almost nothing, and 7337 (31.5%) reported they were unsure. Nearly 1 in 4 transgender adults (5612 of 23 557 individuals [23.8%]) reported having to teach their clinician about transgender people. In total, 3955 respondents (19.4%; 20.8% weighted; 95% CI, 19.2%-22.6%) reported fair or poor self-rated health and 7392 (36.9%; 28.4% weighted, 95% CI, 26.9%-30.1%) met the criteria for severe psychological distress. After adjusting for covariates, compared with individuals who reported their clinician knows almost everything about transgender care, exposure to clinicians with lower perceived levels of knowledge about transgender care was associated with significantly higher odds of fair or poor self-rated health (adjusted odds ratio [aOR] for knowing almost nothing, 2.63; 95% CI, 1.76-3.94; aOR for unsure, 1.81; 95% CI, 1.28-2.56) and severe psychological distress (aOR for knowing almost nothing, 2.33; 95% CI, 1.61-3.37; aOR for unsure, 1.37; 95% CI, 1.05-1.79). Respondents who had to teach a clinician about transgender people had higher odds of reporting fair or poor self-rated health (aOR, 1.67; 95% CI, 1.31-2.13) and severe psychological distress (aOR, 1.49; 95% CI, 1.21-1.83) compared with those who did not.

**Conclusion and Relevance:**

The findings of this cross-sectional study suggest that there is an association between perceived clinician knowledge about transgender people and self-rated health and psychological distress among transgender people. These results highlight the importance of integration and enhancement of transgender health in medical education curriculum as a necessary intervention to improve the health of transgender people.

## Key Points

**Question:**

Is patients’ perceived level of clinician knowledge about transgender care associated with the self-rated health of transgender people?

**Findings:**

In this cross-sectional analysis of 27 715 participants in the 2015 US Transgender Survey, transgender people who had to teach their clinician about transgender people had substantially higher levels of poor self-rated health and severe psychological distress than those who did not have to teach their clinician. Results were similar for transgender people who reported that their clinicians had less transgender-specific knowledge, compared with patients whose clinicians were perceived to have high levels of such knowledge.

**Meaning:**

These findings highlight the importance of integration and enhancement of transgender health in medical education curriculum as a necessary intervention to improve the health of transgender people.

## Teaser

This cross-sectional study examines whether patients’ perceived levels of clinician knowledge of transgender health care are associated with self-rated health status and psychological distress among transgender adults in the US.

## Introduction

Transgender, gender nonbinary, and genderqueer (henceforth, *transgender*) people are more likely to report adverse health outcomes than cisgender people.^1,2,3,4^ For example, an estimated 22% of transgender people estimate their health as fair or poor compared with 18% of the overall US population, and 39% of transgender people currently meet the criteria for severe psychological distress (SPD) compared with 5% of the overall US population.^5^ Long-term stressors, including restrictive policy environments, structural and interpersonal experiences of transphobia, discrimination, stigmatization, and gender minority stress^6^ (“the social stressors specific to transgender and other gender minority people that result from gender-related discrimination, … nonaffirmation of gender identity, internalized transphobia, … community connectedness, and pride”), are important factors contributing to these adverse health outcomes.^7^

Medical education is an important step in addressing these adverse health outcomes and poor clinical experiences of transgender people. Recently graduated physicians interested in providing inclusive care have limited education on the needs of transgender people in their didactic years. In 2014, the Association of American Medical Colleges recognized this gap in medical education.^8^ Their guidelines report that current curricula do not adequately address relevant topics such as gender-affirming care and social determinants of health affecting LGBTQAI (lesbian, gay, bisexual, transgender, queer [or questioning], asexual [or allied], intersex) persons.^8^ Moreover, research regarding LGBTQIA health topics in medical education revealed that medical schools in the US include, on average, only 5 hours of LGBTQIA subject matter.^9^ The limited coverage in medical education is confirmed by learners reporting a lack of competency in several LGBTQIA competencies, including taking a sexual history, discussing sexual orientation or gender identity, and all aspects (ie, medical, social, and mental) of gender-affirming care.^10,11^ Although medical schools have implemented various educational programming ranging from didactic lectures to patient panels, previous review on the content and subsequent quality of LGBTQIA-related material varies greatly across the US.^9^

Little is known regarding clinical perspectives on knowledge or training on transgender-related care, although recent work provides some insight. Among primary care clinicians in an integrated Midwest health system, 85.7% reported being willing to provide routine care to transgender people, and 78.6% reported a wiliness to provide Papanicolaou tests to transgender men.^12^ Overall, 68.8% of primary care physicians reported feeling capable of providing routine care to transgender patients.^12^ When assessing barriers to providing routine care for transgender people, however, 47.9% of primary care clinicians reported a lack of training on transgender health, 37.1% reported a lack of exposure to transgender patients, 32.1% reported a lack of knowledge about transgender care among their staff, and 52.1% reported a lack of familiarity with transition care guidelines.^12^ It remains unclear how these competencies translate into providing care to transgender individuals.

Given recent state-level legislation targeting transgender people and transgender-inclusive health care, concerns about the mental health and well-being of transgender people have heightened. In this study, we examine the association of patients’ perceptions of their clinicians’ knowledge about transgender health with self-rated health (SRH) and SPD among transgender people. Using the 2015 US Transgender Survey (USTS),^5^ the largest and most recently available data set evaluating the lived experiences of transgender people in the US, we investigate 2 hypotheses. First, we hypothesize that greater perceived clinician knowledge regarding transgender health is associated with improved health, including SRH and SPD. Second, we hypothesize that transgender people having to teach their clinicians about transgender people is associated with greater odds of having poor SRH and greater odds of meeting the criteria for SPD.

## Methods

### Study Design

In this cross-sectional study, we performed a secondary data analysis of the 2015 USTS survey.^5^ The protocol for this study was reviewed by the institutional review boards of the University of Alabama at Birmingham, Mississippi State University, and Utah State University and received exempt determinations for human participants research and the need for informed consent because the data were secondary and deidentified. This investigation is reported using the Strengthening the Reporting of Observational Studies in Epidemiology (STROBE) reporting guideline for cross-sectional studies.

### Study Population and Data Source

The 2015 USTS survey was developed and administered online by the National Center for Transgender Equality and includes responses from self-identified transgender, gender nonbinary, and genderqueer individuals aged 18 years and older residing in the US. Although it is a nonprobability sample, with 32 sections and 1140 variables, the USTS is the largest available data collection effort evaluating the lived experiences of self-identified transgender people in the US. Additionally, the USTS has respondents from all 50 states, Washington, DC, American Samoa, Guam, Puerto Rico, and US military bases overseas.^5^ Since 2019, the data set has been archived at the Inter-University Consortium for Political and Social Research at the University of Michigan.^13^ The USTS protocol was approved by the University of California, Los Angeles institutional review board.^5^ Our analysis was based on subsamples of respondents for SRH models and SPD models, both representing the number of respondents with complete responses on all variables used.

### Outcomes

We examined 2 binary outcomes. First, SRH was dichotomized as poor or fair vs excellent, very good, or good, following the analytic strategy of prior studies of SRH.^14,15,16,17,18,19,20^ The second outcome was SPD, defined as scoring a validated threshold of 13 or greater on the Kessler Psychological Distress Scale.^21,22^

### Exposures

The primary exposure of interest was patients’ perceived clinician knowledge about transgender health care. This exposure is a composite measure built from responses to 3 questions: (1) “Thinking about the doctor or provider you go to for your trans-related health care (such as hormone treatment), how much do they know about providing health care for trans people?” (2) “Do you see your trans-related provider for routine care?” and (3) “How much does your routine health care provider (who you see for physicals, flu, diabetes, etc.) know about health care for trans people?” We used the level of perceived knowledge of clinicians who provided transgender-specific care for respondents who do not have a routine care clinician or who use their transgender-related care clinicians for routine care. We used the level of knowledge of routine care clinicians for respondents who only have a routine care clinician or who have a different clinician for transgender-related care vs routine care. In secondary analysis (not shown), we considered separate models for transgender-related care clinicians and routine care clinicians. Ultimately, we combined transgender-related care and routine care clinician knowledge so that we could retain a larger analytic sample while capturing the overall experience with medical clinicians. Variable attributes included the patients’ perception that the clinician (1) knows almost everything [about transgender-related care], (2) knows most things [about transgender-related care], (3) knows some things [about transgender-related care], (4) knows almost nothing [about transgender-related care], and (5) I am not sure.

In the second set of models, the primary exposure was whether respondents had taught their clinician about transgender-related care, a dichotomous measure in which respondents answered no (0) or yes (1) to the statement, “I had to teach my doctor or other health care provider about trans people so that I could get appropriate care” in the past year.

### Covariates

Sociodemographic covariates examined include gender identity (transgender woman, transgender man, and gender nonbinary or genderqueer), age (18-24 years, 25-44 years, 45-64 years, and ≥65 years), marital status (married or cohabitating, never married, divorced, and widowed), and race and ethnicity. Race and ethnicity were determined by self-report, and were categorized as biracial or multiracial (indicating respondents who selected 2 or more racial and ethnic categories), Latinx or Hispanic, non-Hispanic American Indian or Alaska Native, non-Hispanic Asian American, non-Hispanic Black or African American, non-Hispanic Middle Eastern or North African, non-Hispanic Native Hawaiian or other Pacific Islander, and non-Hispanic White. Race and ethnicity were included to account for racial and ethnic disparities observed among the study sample.^5^

Socioeconomic covariates included education level (less than high school, high school diploma or general educational development, some college, associate’s degree, bachelor’s degree, and graduate or professional degree), employment status (employed or unemployed), and health insurance status (insured or uninsured). A control variable for using the same clinician was included, which measured whether the same clinician provided transgender-related care and routine care for respondents.

### Statistical Analysis

All analyses were conducted using Stata statistical software version 17 (StataCorp)^23^ using complex survey design (svy) procedures and weights created by the USTS that correct for purposive nonprobability sampling bias on race and ethnicity, age, and education level. Weighted descriptive statistics were calculated and are presented for variables included in models. Due to the dichotomous nature of both outcome variables, binary logistic models were estimated to test whether exposures of interest were associated with outcomes, and were expressed in adjusted odd ratios (aORs). The aORs with 95% CIs and 2-sided *P* values are reported with a *P* < .05 threshold for significance. Respondents with missing data for exposure and outcome variables were excluded without compensatory methods.^24^ Data were analyzed from February to November 2022.

## Results

The 2015 USTS survey included responses from 27 715 transgender, gender nonbinary, and genderqueer individuals (9238 transgender women [33.3%; 55.1% weighted; 95% CI, 53.4%-56.7%], 22 658 non-Hispanic White individuals [81.8%; 65.6% weighted; 95% CI, 63.7%-67.5%], and 4085 individuals aged 45-64 years [14.7%; 33.8% weighted; 95% CI, 32.0%-35.5%]). A total of 19 463 respondents (70.2%; 52.0% weighted; 95% CI, 50.4%-53.7%) reported their marital status as never married, 26 809 (96.7%; 85.6% weighted; 95% CI, 83.4%-87.5%) had a high school diploma or higher, 24 211 (87.4%; 85.1% weighted; 95% CI, 83.9%-86.3%) had health insurance, and 18 000 (65.0%; 59.1% weighted; 95% CI, 57.3%-60.9%) were employed. The subsamples for this study included 20 381 respondents for SRH models and 20 037 respondents for SPD models. Almost one-half of respondents (12 655 respondents [45.7%]) reported not having a transgender-related care clinician, 4119 respondents (14.9%) lacked a routine care clinician, and 7465 respondents (26.9%) used the same clinician for transgender-related and routine care.

Of 23 318 individuals who answered questions regarding their perceptions of their clinicians’ level of knowledge, 5732 (24.6%) reported their clinician knows almost everything about transgender care, 4083 (17.5%) reported their clinician knows most things, 3446 (14.8%) reported their clinician knows some things, 2680 (11.5%) reported their clinician knows almost nothing, and 7337 (31.5%) reported they were unsure. Table 1 provides the results of the descriptive analysis. The 2 outcomes, SRH and SPD, varied by perceived level of clinician knowledge. The proportion of respondents reporting fair or poor SRH and SPD increased with lower levels of clinician knowledge. Nearly a quarter of respondents (5,612 respondents [23.8%]) in the study sample had to teach their clinician about transgender-related care. Among 5,612 respondents who had to teach their clinician about transgender care, 1341 respondents (23.9%) reported fair or poor SRH and 2209 (40.1%) met the criteria for SPD.

**Table 1.** Respondent Demographics and Patients’ Perceived Level of Clinician Knowledge of Transgender Health Care^a^

Characteristic	Respondents, No. (%)						
	Patients perceived level of clinician knowledge of transgender care (n = 23 318)					Had to teach clinician about transgender care (n = 23 557)	
	Knows almost everything (n = 5732)	Knows most things (n = 4083)	Knows some things (n = 3446)	Knows almost nothing (n = 2680)	I’m not sure (n = 7377)	No (n = 17 945)	Yes (n = 5612)
Self-rated health							
Excellent, very good, or good	5039 (87.9)	3511 (86.0)	2793 (81.1)	1839 (68.6)	5660 (68.6)	14582 (81.3)	4268 (76.1)
Fair or poor	690 (12.0)	569 (13.9)	653 (18.9)	838 (31.3)	1715 (23.2)	3355 (18.7)	1341 (23.9)
Kessler Psychological Distress Scale score indicating severe psychological distress							
No	4163 (72.6)	2795 (68.5)	2231 (64.7)	1277 (47.6)	4040 (54.8)	11229 (62.6)	3294 (58.7)
Yes	1452 (25.3)	1212 (29.7)	1156 (33.5)	1355 (50.6)	3210 (43.5)	6421 (35.8)	2209 (39.4)
Sees same clinician for transgender-related and routine care							
No	2750 (48.0)	2006 (49.1)	2001 (58.1)	2128 (79.4)	6872 (93.2)	12831 (71.5)	3402 (60.6)
Yes	2965 (51.7)	2067 (50.6)	1440 (41.8)	540 (20.1)	370 (5.0)	5005 (27.9)	2190 (39.0)
Gender identity							
Transgender woman	2827 (49.3)	1940 (47.5)	1383 (40.1)	790 (29.5)	1256 (17.0)	5990 (33.4)	2082 (37.1)
Transgender man	2238 (39.0)	1544 (37.8)	1257 (36.5)	783 (29.2)	1166 (15.8)	4785 (26.7)	2240 (39.9)
Gender nonbinary or genderqueer	639 (11.1)	575 (14.1)	766 (22.2)	1049 (39.1)	4470 (60.6)	6608 (36.8)	1275 (22.7)
Race and ethnicity							
American Indian or Alaska Native	58 (1.0)	39 (1.0)	58 (1.7)	59 (2.2)	59 (0.8)	175 (1.0)	106 (1.9)
Asian American	138 (2.4)	84 (2.1)	87 (2.5)	71 (2.6)	221 (3.0)	489 (2.7)	110 (2.0)
Biracial or multiracial^b^	264 (4.6)	192 (4.7)	176 (5.1)	170 (6.3)	418 (5.7)	929 (5.2)	306 (5.5)
Black or African American	219 (3.8)	107 (2.6)	77 (2.2)	61 (2.3)	179 (2.4)	502 (2.8)	149 (2.7)
Hispanic or Latinx	306 (5.3)	179 (4.4)	171 (5.0)	128 (4.8)	421 (5.7)	917 (5.1)	264 (4.7)
Middle Eastern or North African	20 (0.3)	19 (0.5)	DS^c^	DS^c^	DS^c^	77 (0.4)	32 (0.6)
Native Hawaiian or other Pacific Islander	17 (0.3)	11 (0.3)	DS^c^	DS^c^	DS^c^	41 (0.2)	11 (0.2)
White	4710 (82.2)	3452 (84.5)	2856 (82.9)	2167 (80.9)	6039 (81.9)	14815 (82.6)	4634 (82.6)
Age, y							
18-44	4358 (76.0)	3215 (78.7)	2631 (76.3)	2209 (82.4)	6355 (86.1)	14579 (81.2)	4560 (81.3)
45 to 64	1156 (20.2)	743 (18.2)	690 (20.0)	394 (14.7)	798 (10.8)	2754 (15.3)	920 (16.4)
≥65	218 (3.8)	125 (3.1)	125 (3.6)	77 (2.9)	224 (3.0)	612 (3.4)	132 (2.4)
Marital status							
Married or cohabitating	1278 (22.3)	865 (21.2)	789 (22.9)	489 (18.2)	1185 (16.1)	3429 (19.1)	1163 (20.7)
Never married	3555 (62.0)	2623 (64.2)	2133 (61.9)	1911 (71.3)	5690 (77.1)	12532 (69.8)	3660 (65.2)
Divorced	748 (13.0)	484 (11.9)	437 (12.7)	229 (8.5)	378 (5.1)	1603 (8.9)	651 (11.6)
Widowed	66 (1.2)	32 (0.8)	31 (0.9)	22 (0.8)	51 (0.7)	157 (0.9)	43 (0.8)
Education level							
Less than high school	120 (2.1)	75 (1.8)	58 (1.7)	107 (4.0)	342 (4.6)	562 (3.1)	137 (2.4)
High school diploma or general educational development	570 (9.9)	336 (8.2)	305 (8.9)	353 (13.2)	1172 (15.9)	2190 (12.2)	503 (9.0)
Some college	1861 (32.5)	1349 (33.0)	1200 (34.8)	1074 (40.1)	3201 (43.4)	6839 (38.1)	1973 (35.2)
Associate’s degree	561 (9.8)	362 (8.9)	313 (9.1)	235 (8.8)	523 (7.1)	1467 (8.2)	512 (9.1)
Bachelor’s degree	1671 (29.2)	1269 (31.1)	924 (26.8)	590 (22.0)	1497 (20.3)	4537 (25.3)	1550 (27.6)
Graduate or professional degree	949 (16.6)	692 (16.9)	646 (18.7)	321 (12.0)	642 (8.7)	2350 (13.1)	937 (16.7)
Has health insurance							
No	475 (8.3)	355 (8.7)	295 (8.6)	282 (10.5)	474 (6.4)	1677 (9.3)	505 (9.0)
Yes	5250 (91.6)	3727 (91.3)	3147 (91.3)	2395 (89.4)	6878 (93.2)	16235 (90.5)	5102 (90.9)
Employment							
Employed	4141 (72.2)	2938 (72.0)	2370 (68.8)	1600 (59.7)	4215 (57.1)	11633 (64.8)	3790 (67.5)
Unemployed	1566 (27.3)	1126 (27.6)	1064 (30.9)	1070 (39.9)	3121 (42.3)	6232 (34.7)	1799 (32.1)

Abbreviation: DS, data suppressed.

^a^ Column percentages may not add up to 100% because missing data are not displayed.

^b^ Biracial or multiracial indicate respondents who selected 2 or more racial and ethnic categories.

^c^ Data suppressed for privacy purposes.

Table 2 and Table 3 present the results of the regression analysis. After adjusting for sociodemographic and socioeconomic covariates, exposure to a clinician with lower perceived levels of knowledge about transgender-related care was associated with higher odds of poor or fair SRH and SPD. Respondents who reported their clinician knew some things (aOR, 1.45; 95% CI, 1.09-1.93; *P* = .01), knew almost nothing (aOR, 2.63; 95% CI, 1.76-3.94; *P* < .001), and were not sure how much their clinician knew about transgender-related care (aOR, 1.81; 95% CI, 1.28-2.56; *P* < .001) were more likely to report poor or fair SRH compared with respondents who reported their clinician knew almost everything about transgender-related care. Similarly, respondents who reported their clinician knew some things (aOR, 1.35; 95% CI, 1.07-1.70; *P* = .01), knew almost nothing (aOR, 2.33; 95% CI, 1.61-3.37; *P* < .001), and were not sure how much their clinician knew (aOR, 1.37; 95% CI, 1.05-1.79; *P* = .02) were more likely to report SPD compared to respondents who reported their clinician knew almost everything about transgender-related care. Respondents who had to teach a clinician about transgender-related care had higher odds of reporting poor or fair SRH (aOR, 1.67; 95% CI, 1.31-2.13; *P* < .001) and SPD (aOR, 1.49; 95% CI, 1.21-1.83; *P* < .001) compared with respondents who did not have to teach their clinician about transgender-related care (Table 3).

**Table 2.** Association of Patients’ Perception of Clinician Knowledge About Transgender Care With Poor or Fair SRH and SPD^a^

Patients’ perception of clinician’s knowledge about transgender care^b^	SRH^c^		SPD^d^	
	aOR (95% CI)	*P* value	aOR (95% CI)	*P* value
Knows most things	1.18 (0.85-1.64)	.33	1.28 (0.97-1.67)	.08
Knows some things	1.45 (1.09-1.93)	.01	1.35 (1.07-1.70)	.01
Knows almost nothing	2.63 (1.76-3.94)	<.001	2.33 (1.61-3.37)	<.001
I’m not sure	1.81 (1.28-2.56)	<.001	1.37 (1.05-1.79)	.02

Abbreviations: aOR, adjusted odds ratio, SPD, severe psychological distress, SRH, self-rated health.

^a^ Adjusted for same clinician, gender identity, race and ethnicity, age, marital status, education level, insurance status, employment status.

^b^ Reference for knowledge about transgender care is knows almost everything.

^c^ Refers to poor or fair SRH vs good, very good, or excellent SRH (N = 20 381).

^d^ SPD is defined as a score of 13 or higher on the Kessler Psychological Distress Scale (N = 20 037).

**Table 3.** Association of Having to Teach Clinician About Transgender People with Poor/Fair SRH and SPD^a^

Outcome	aOR (95% CI)	*P* value
Poor or fair self-rated health^c^	1.67 (1.31-2.13)	<.001
Severe psychological distress^d^	1.49 (1.21-1.83)	<.001

Abbreviations: aOR, adjusted odds ratio, SPD, severe psychological distress, SRH, self-rated health.

^a^ Adjusted for same clinician, gender identity, race and ethnicity, age, marital status, education level, insurance status, employment status.

^b^ Reference for knowledge about transgender care is knows almost everything.

^c^ Refers to poor or fair SRH vs good, very good, or excellent SRH (N = 20 381).

^d^ SPD is defined as a score of 13 or higher on the Kessler Psychological Distress Scale (N = 20 037).

## Discussion

To our knowledge, this is the first large-scale study to demonstrate an association of perceived clinician knowledge about transgender people and transgender health with health outcomes of transgender people. In this cross-sectional study, we found that greater perceived clinician knowledge was associated with higher odds of reporting better SRH and lower odds of reporting SPD among transgender people. Nearly 1 in 4 transgender people reported having to teach their clinician about transgender people, and more than 1 in 2 transgender people reported that their clinician appeared to know almost nothing about health care for transgender people.

This perceived lack of knowledge about transgender people and health care for transgender people among clinicians was negatively associated with SRH and SPD. Although this association remained after controlling for a host of covariates, we stop short at suggesting the education of clinicians alone will improve the mental and overall health of transgender people. Importantly, other factors contribute to the adverse health outcomes of transgender patients. Restrictive state policy environments are associated with poor health and less access to care for transgender people.^25,26,27,28^ Experiences of discrimination, stigmatization, and gender minority stress outside the health care context are also associated with negative health outcomes.^7^ Among primary care clinicians in an integrated health care system in the US Midwest, increasing hours of transgender health care education was not significantly associated with knowledge about transgender health care.^29^ However, transphobia was significantly associated with clinician knowledge, suggesting that increased education alone may be insufficient in addressing the lack of knowledge about transgender care among health care clinicians.^29^

Nevertheless, the integration of education surrounding transgender people and transgender health disparities is an important step to address ongoing health disparities transgender populations face. Patients benefit from clinicians trained to understand the health disparities experienced by transgender people and to use best communication practices for these patients. Kattari et al^30^ explored the association of transgender individuals seeking inclusive clinicians with changes in mental health outcomes. Their results indicate that transgender individuals who saw a gender-inclusive clinician and had preexisting mood disorders showed almost a 50% decrease in suicidality, defined as having suicidal thoughts within the past year.^30^ Ultimately, incorporating more LGBTQAI health topics and considerations on social determinants of health into the medical curriculum provides positive outcomes for learners and future patients.^31,32^

### Strengths and Limitations

The current study has several strengths worth highlighting. First, we used the largest available data set evaluating the lived experiences and health of transgender, gender nonbinary, and genderqueer people in the US.^5^ The data included respondents from all 50 US states, Washington, DC, and American territories and military bases overseas. Second, this study is the first, to our knowledge, to examine the association of perceived clinician knowledge about transgender health with SRH and SPD among transgender people. Examining this association allows us to better understand the roles that medical education and clinician training play in the everyday health status of transgender people. Finally, we included a host of sociodemographic and socioeconomic covariates in our models, which the literature indicate are directly associated with health.^5^ Although these covariates slightly attenuated effect sizes, statistical significance remained in adjusted models.

The study is limited by its cross-sectional study design as well as nonprobability sampling technique. The USTS lacks racial and ethnic diversity with the majority of the sample being White; Black and Latinx populations are largely underrepresented. As a result, the weighting procedure used by the USTS presents concerns, primarily because the weighting procedures are based on the general US population; it is possible that the distribution of race and ethnicity, age, and education level would be different in the transgender population than the general US population.^33^ In addition, our primary exposure variable measures the individual’s perceptions of their clinician’s knowledge about transgender health rather than the clinician’s self-reporting of their education and training. However, our results indicate an association of diminished health status with perceived clinician knowledge, indicating that perception does matter. Additionally, our models include whether a transgender person reported having to teach their clinician about transgender people, which is potentially a more definitive measure of clinician knowledge because this variable is most likely dependent on whether a respondent needed their clinician to know new information or act differently. For example, respondents had to inform their clinicians about the expected protocols related to transgender health, including monitoring testosterone levels and risk of cardiac disease among transgender men.

## Conclusions

In this cross-sectional study of transgender, nonbinary, and genderqueer adults, we found that patients’ perceived levels of knowledge clinicians had about transgender people and providing transgender health care was associated with health outcomes of transgender people. Our results demonstrate that transgender people who had to teach their clinicians about transgender people and who reported their clinicians had lower levels of knowledge about transgender health care were at significantly higher odds of reporting fair or poor SRH and were at significantly higher odds of meeting the criteria for SPD. These findings provide empirical evidence to support the integration and enhancement of transgender health care and the impacts of gender identity in the medical education curriculum as a necessary intervention to improve the health of transgender, gender nonbinary, and genderqueer people.

## Notes

Supplement 1.
Data Sharing Statement
