# Supplementary material for: Patients’ Perceived Level of Clinician Knowledge of Transgender Health Care, Self-rated Health, and Psychological Distress Among Transgender Adults
Source: JAMA Netw Open. 2023 May 25;6(5):e2315083. doi: 10.1001/jamanetworkopen.2023.15083 (PMC10214034; doi:10.1001/jamanetworkopen.2023.15083)
Supplement: Supplement 1. — Data Sharing Statement [file jamanetwopen-e2315083-s001.pdf]

Data Sharing Statement

Miller. Patients' Perceived Level of Clinician Knowledge of Transgender Health Care, Self-Rated Health, and Psychological Distress Among Transgender Adults. *JAMA Netw Open*. Published May 25, 2023. doi:10.1001/jamanetworkopen.2023.15083

Data
Data available: No

Additional Information
Explanation for why data not available: The data used in this article are archived at ICPSR and it is restricted data. For more information, see the following: Sandy E. James, NCTE (U.S.); Jody Herman, NCTE (U.S.); Mara Keisling, NCTE (U.S.); Lisa Mottet, NCTE (U.S.); and Ma'ayan Anafi, NCTE (U.S.). https://doi.org/10.3886/ICPSR37229.v1
